# Global Cardiac Surgery in Brazil: A Call to Action

**DOI:** 10.21470/1678-9741-2023-0408

**Published:** 2024-05-13

**Authors:** Matheus Daniel Faleiro, Miguel Godeiro Fernandez, Kawanna Izabella Buzzo Feitosa, Dominique Vervoort

**Affiliations:** 1 International Student Surgical Network (InciSioN), Belo Horizonte, Minas Gerais, Brazil; 2 Faculdade de Medicina, Universidade Federal de Minas Gerais, Belo Horizonte, Minas Gerais, Brazil; 3 Escola Bahiana de Medicina e Saúde Pública, Salvador, Bahia, Brazil; 4 Faculdade de Medicina, Universidade Positivo, Curitiba, Paraná, Brazil; 5 Division of Cardiac Surgery, University of Toronto, Toronto, Ontario, Canada; 6 Institute of Health Policy, Management and Evaluation, University of Toronto, Toronto, Ontario, Canada

**Keywords:** Brazil, Cardiac Surgical Procedures, Health Care Outcome Assessment, Heath Services Accessibility, Healthcare Disparities

## Abstract

Global Cardiac Surgery is an innovative initiative with a focus on improving health outcomes and achieving healthcare equity for individuals worldwide affected by cardiac surgical conditions or in need of cardiac surgical care. Considering the existing disparities in access to cardiac surgery and the substantial burden of cardiac conditions amenable to surgical procedures in Brazil, it is imperative to support and scale Global Cardiac Surgery initiatives and leave no Brazilian patient behind. Here, we advocate for national initiatives within this field and highlight opportunities and challenges to support their development.

## INTRODUCTION

**Table:** 

Abbreviations, Acronyms & Symbols	
CHD	= Congenital heart disease
GCS	= Global Cardiac Surgery
HICs	= High-income countries
IHD	= Ischemic heart disease
RHD	= Rheumatic heart disease
SAO	= Surgical, anesthetic, and obstetric

Five billion people lack access to safe, timely, and affordable surgical, anesthetic, and obstetric (SAO) care when required. Access is worst in lowand middle-income countries, where nine of ten people cannot access emergency and essential surgical care^[1]^. In 2015, the Lancet Commission on Global Surgery brought worldwide attention to the field of global surgery, which has since emerged as a rapidly growing multidisciplinary field. Although definitions may vary, global surgery seeks to increase access to SAO services in a safe, equitable, and affordable manner. In recent years, many surgical subspecialties have followed the broader global surgery momentum^[2]^.

## COMMENTS

### Global Cardiac Surgery

The field of Global Cardiac Surgery (GCS) emerged in this context and can be defined as *“an area for study, research, practice, and advocacy that places priority on improving health outcomes and achieving health equity for all people worldwide who are affected by cardiac surgical conditions or have the need for cardiac surgical care”*^[2]^. As such, this movement aims to integrate cardiac surgery into evolving health systems, shifting from past short-term humanitarian missions that characterized the majority of historical global cardiac efforts in the global health context towards sustainable efforts across the care continuum and society. In just five years, GCS has expanded rapidly, and all major European, North American, Latin American, and African societies have successfully incorporated GCS sessions into their annual meetings, research efforts have shone further light on the topic, trainee efforts culminated in the establishment and growth of the Global Cardiac Surgery Initiative, and the first textbook on this topic has been published^[3]^.

### Cardiac Surgery in Brazil

Despite the aforementioned progress in GCS, gaps prevail in Brazil. Zilla et al. (2018)^[4]^ estimated that Brazil has 10.4 cardiac surgeons per million people, higher than the average of high-income countries (HICs)^[5]^, but raised concerns regarding the heterogeneity in the availability of cardiac surgery services within the Brazilian territory, with the South and Southeast regions possessing a disproportionate share of the 337 national cardiac centers. A deeper concern arises when we examine the geographical dispersion of the cardiovascular surgery workforce in Brazil. Data from the last Demografia Médica Brasileira^[6]^ shows that there is a density of 1.20 cardiovascular surgeons per 100,000 population in the country. However, significant regional disparities are present at the state level: the density of cardiovascular surgeons stands at 1.75 per 100,000 population in the state of São Paulo, whereas in the state of Roraima, the density is as low as 0.16 per 100,00 population. This raises significant concerns related to access to cardiovascular procedures on Brazilian territory.

Among the cardiovascular diseases that most require procedures, rheumatic heart disease (RHD), congenital heart disease (CHD), and ischemic heart disease (IHD) are most prevalent in the country. Brazil registers 30,000 cases of acute rheumatic fever annually, with an RHD incidence of seven cases per 1,000 school-age children. In comparison, HICs exhibit an incidence rate of only 0.4 per 1,000 children within the same age group, primarily affecting children from immigrant or marginalized populations^[7]^. This reality accompanies increasing mortality annually with important underreporting, in which RHD sequelae are responsible for one-third of the Brazilian cardiovascular surgery volume^[7]^. RHD is a disease linked to poverty and social factors, such as overcrowded housing. Its prevalence within the country suggests a health system that still fails to raise public health and parental awareness and also fails to detect and intervene before RHD occurs, which leads to late diagnosis and development of severe cases with cardiovascular implications^[8]^.

CHD is the most common congenital anomaly and occurs in one in 100 live births globally. In Brazil, CHD is the second main cause of death in children below one year of age^[9]^. Although crucial strategies have been adopted for neonatal screening in Brazil, such as mandatory pulse oximetry testing since 2014, health services are still not totally equipped with basic resources to manage this disease, and higher complexity units require investment in technology and trained professionals. In that regard, some positive initiatives can be mentioned, such as the partnership between Children’s HeartLink and Brazilian centers. This partnership has resulted in notable enhancements in the quality of care for children with CHD in the country, a strengthened multidisciplinary approach, and an expanded number of children receiving the necessary treatment^[10]^. Another reality is CHD in adults, a growing burden for health systems worldwide, also observed within the Brazilian context. In children with less complex but still notable CHD that allows them to survive into adulthood without intervention, this may reflect a deficiency in timely detection and treatment of CHD, eventually producing persistent structural abnormalities that compromise quality of life later in life^[11]^. In addition, there is also a need to address care for adults with CHD who were operated on and now require lifelong care for their CHD and related conditions.

Concerning IHD, Oliveira et al. (2020)^[12]^ indicate that it is still the major cause of cardiovascular death in Brazil, reaching a proportion of 32.1% of total cardiovascular diseases. In recent years, there has been a notable increase in the volume of coronary artery bypass grafting procedures within the Brazilian public health system - the Sistema Único de Saúde. A significant portion of these surgeries, approximately half, is conducted in the wealthier Southeast region, with only 2.8% of these procedures performed in the North region^[13]^. Disparities also exist when considering regional mortality rates associated with these procedures, since a previous study has shown a higher risk of mortality in the Southern, Northern, and Central-West regions of the country^[13]^.

### Supporting Global Cardiac Surgery Initiatives in Brazil

Immediate interventions are vital to change the current scenario, and the first one is to ensure the provision of adequate training to cardiovascular surgery residents in Brazil. Although cardiac surgery residency has faced major changes in 2018, historically, the number of residency positions in this specialty has been 228 per year in Brazil, and there is a recent debate by the Sociedade Brasileira de Cirurgia Cardiovascular on whether to decrease the number of these positions^[14]^. Concerns naturally arise regarding the prudence of this decision, given that the conclusion of the epidemiological transition in the country will result in a gradual disappearance of RHD and its replacement with degenerative/lifestyle diseases. This transition will lead to a fourto fivefold increase in the demand for cardiac surgery once completed, necessitating a greater need for cardiovascular surgeons in the country^[4]^.

Research in Brazilian cardiovascular surgery remains at a level far below its real potential, primarily due to substantial limitations in research funding and dedicated research time^[15]^. Unlike HICs, where multiple institutions sponsor research initiatives, funding opportunities in Brazil are primarily provided by governmental entities, and there is a concerning trend of decreasing resources over time^[16]^. Additionally, surgeons often do not have dedicated time for research as a part of their training, a practice common in HICs that is essential to form future academic cardiovascular surgeons. To address this issue, it is imperative to establish initiatives that support research for Brazilian cardiovascular trainees and surgeons, with a focus on creating an environment permissive to the development of GCS research initiatives in Brazil.

By establishing public policies, the Brazilian government can play a pivotal role in ensuring that a broader segment of the population can access the necessary cardiac surgical procedures, which should ideally be available for free and at any time through the public healthcare system. These policies should encompass not only the expansion of the specialized workforce but also the enhancement of infrastructure and facilities crucial for the delivery of cardiac surgery, with a particular focus on rural areas where specialized centers are frequently lacking^[17]^. Incorporating cardiac surgery into a potential future Brazilian national surgical, obstetric, and anesthesia plan is also essential to ensure that these life-saving procedures become easily accessible to all Brazilian citizens.

Finally, advocacy efforts are also needed to enhance the recognition of GCS' importance in the Brazilian context. Multiple organizations have been working on advocacy efforts in the Latin America region, such as the Latin American Association of Cardiac and Endovascular Surgery, the Global Cardiac Surgery Initiative, and International Student Surgical Network Brazil. Promotion via social media could potentially serve as a means to accelerate this goal, taking into account the insights gained from previous global surgery initiatives in Brazil^[18]^. Additionally, the engagement of professional societies in this movement is also a vital step in establishing the groundwork for GCS initiatives in the country.

## CONCLUSION

The need to integrate GCS into the Brazilian healthcare framework is evident, given the significant disparities in access to cardiovascular procedures and the substantial burden of RHD, CHD, and IHD in the country. To achieve this aim, raising awareness about this field, enhancing cardiovascular surgical training, and supporting research initiatives are imperative. Furthermore, government commitment to policy development and resource allocation is crucial to ensuring equitable access.
